# Prediction of Spinal Pain Intensity from Perception-Dependent Stimulation Thresholds and Patient Characteristics: A Regression and Machine Learning Approach

**DOI:** 10.3390/diagnostics16203403

**Published:** 2026-10-20

**Authors:** Kübra Okuyucu, Aybike Şenel, Ahmet Gülünay, Murat Yücel

**Affiliations:** 1 Department of Physiotherapy and Rehabilitation, Faculty of Health Sciences, Amasya University, 05100 Amasya, Türkiye; aybike.senel@amasya.edu.tr; 2 Department of Physiotherapy and Rehabilitation, Graduate Education Institute, Karabük University, 78050 Karabük, Türkiye; fztahmetgulunay@gmail.com; 3 Department of Biostatistics, Faculty of Medicine, Eskişehir Osmangazi University, 26480 Eskişehir, Türkiye; murat.yucel@amasya.edu.tr; 4 Department of Medical Services and Techniques, Sabuncuoğlu Şerefeddin Health Services Vocational School, Amasya University, 05100 Amasya, Türkiye

**Keywords:** back pain, electromyography, machine learning, pain measurement, prediction algorithms

## Abstract

**Background and Objective**: Spinal pain is highly prevalent, yet clinically accessible tools for pain quantification remain limited. This study evaluated whether machine learning (ML) algorithms using StimaWELL 120MTRS-derived scores alongside demographic variables can predict self-reported spinal pain intensity and compared their performance against linear mixed model (LMM)-based associations. **Methods**: Seventy-eight adults with spinal pain (44 female, 34 male; mean age 43.08 ± 11.50 years) were assessed under two electrode configurations: opposing electrode (OE) and adjacent electrode (AE). Self-reported pain was measured using the Numeric Pain Rating Scale (NPRS). Repeated measures correlation, intraclass correlation coefficients (ICC), LMM, and eight ML algorithms were applied to evaluate predictive capacity. **Results**: Repeated measures correlations between StimaWELL scores and NPRS were negligible and non-significant (OE: r = −0.003, *p* = 0.970; AE: r = 0.015, *p* = 0.876), with ICC values indicating poor agreement (OE: 0.364; AE: 0.452). LMM revealed no statistically significant fixed effects for either configuration. Despite this, ML algorithms achieved moderate predictive performance, with Extra Trees and CatBoost yielding the highest tolerance-based accuracy (up to 73–74% for AE-based models). The AE StimaWELL score was the dominant predictor across feature importance analyses, followed by body weight and height. **Conclusions**: ML algorithms extracted the predictive signal from StimaWELL 120MTRS data where LMM found none, highlighting nonlinear relationships between device-derived scores and pain intensity. AE configuration demonstrated superior predictive performance. These findings suggest that StimaWELL 120MTRS may provide complementary quantitative information in spinal pain assessment, pending external validation in larger, diverse populations.

## 1. Introduction

Spinal pain is a leading cause of disability and healthcare expenditure worldwide. In 2021, low back pain accounted for 7.7% of all years lived with disability, ranking first globally, while neck pain ranked 15th [1,2]. Its multifactorial etiology makes diagnosis and effective treatment challenging, and accurate assessment remains central to clinical decision-making [3,4].

Pain is inherently subjective, and self-report measures alone are insufficient to fully capture its complexity, particularly in chronic spinal conditions where psychological factors interact with pain perception [5,6]. Quantitative assessment methods offer a more reliable means of determining pain severity and its functional consequences and may help avoid unnecessary imaging or surgical intervention [7]. Quantitative sensory testing, using electrical, thermal, or chemical stimuli to measure sensory system responses, plays an important role in determining pain thresholds, pain intensity, and alterations in sensory sensitivity by measuring sensory responses of the nervous system [8,9].

The StimaWELL 120MTRS is a whole-spine electrical stimulation system that delivers modulated electrical currents through a 12-channel paraspinal mat extending from the cervical to sacral regions [10,11]. Before treatment, the system performs a standardized calibration in which stimulation intensity is progressively increased until the participant reports a “strong but non-painful sensation” at each electrode pair, generating segmental stimulation-threshold scores [11]. These scores are therefore perception-dependent rather than direct measures of pain or muscle activity and may be influenced by sensory processing, tissue characteristics, and neuromuscular responses. This provides a rationale for investigating their relationship with self-reported pain intensity, while recognizing that the two constructs are not equivalent. Unlike surface electromyography, which records muscle electrical activity, or conventional quantitative sensory testing evaluating defined sensory or pain thresholds [12], StimaWELL calibration provides a standardized measure of tolerance to paraspinal electrical stimulation. Importantly, the system can also use these measurements within an AI (artificial intelligence)-assisted therapeutic program, linking the calibration process to individualized whole-spine electrotherapy [10,11,13,14]. Recent evidence has demonstrated improvements in pain and disability following StimaWELL treatment in individuals with chronic low back pain [11]. However, whether the device-derived calibration scores themselves reflect or predict pain intensity remains unclear. The present study addresses this gap by examining whether scores obtained using opposing-electrode (OE) and adjacent-electrode (AE) configurations can predict self-reported spinal pain intensity.

Machine learning (ML) approaches are showing growing promise in pain assessment and management. Studies have demonstrated that AI-based systems can interpret neurophysiological signals for pain intensity evaluation [15,16], although challenges remain regarding clinical workflow integration, model interpretability, and cross-population validation [17,18,19]. The StimaWELL 120MTRS device, which combines whole-body electrical muscle stimulation with AI-assisted analysis to generate individualized musculoskeletal assessment protocols, represents one such tool with potential clinical utility in spinal pain evaluation [10,11,13,14].

This study aimed to assess individuals with spinal pain using the StimaWELL 120MTRS and to investigate whether device-derived scores can predict self-reported pain intensity. Both Linear Mixed Model-based associations and ML algorithms were applied to evaluate the predictive capacity of StimaWELL measurements, with the aim of determining whether this AI-assisted calibration protocol may serve as a viable assessment tool in spinal pain.

## 2. Materials and Methods

### 2.1. Study Design

This is an exploratory cross-sectional study that was conducted between November 2025 and January 2026. This study was executed in accordance with the guidelines of the Declaration of Helsinki and was approved by the Non-Interventional Clinical Research Ethics Committee of Amasya University (Protocol Code: E-76988455-050.01-263355 and date of approval: 23 May 2025; Trial registration number at ClinicalTrials.gov: NCT07244237, prospectively registered). After explaining the study procedure, written and verbal informed consent was obtained from all the participants. Each assessment was performed only once by the same researcher. The analysis dataset was structured at the observation level, with each participant being assessed on multiple occasions, yielding a total of 266 observations from 78 participants. This study was reported in accordance with the STROBE guidelines (Supplementary Materials, Checklist S1).

### 2.2. Participant Recruitment and Selection

**Figure 1:**
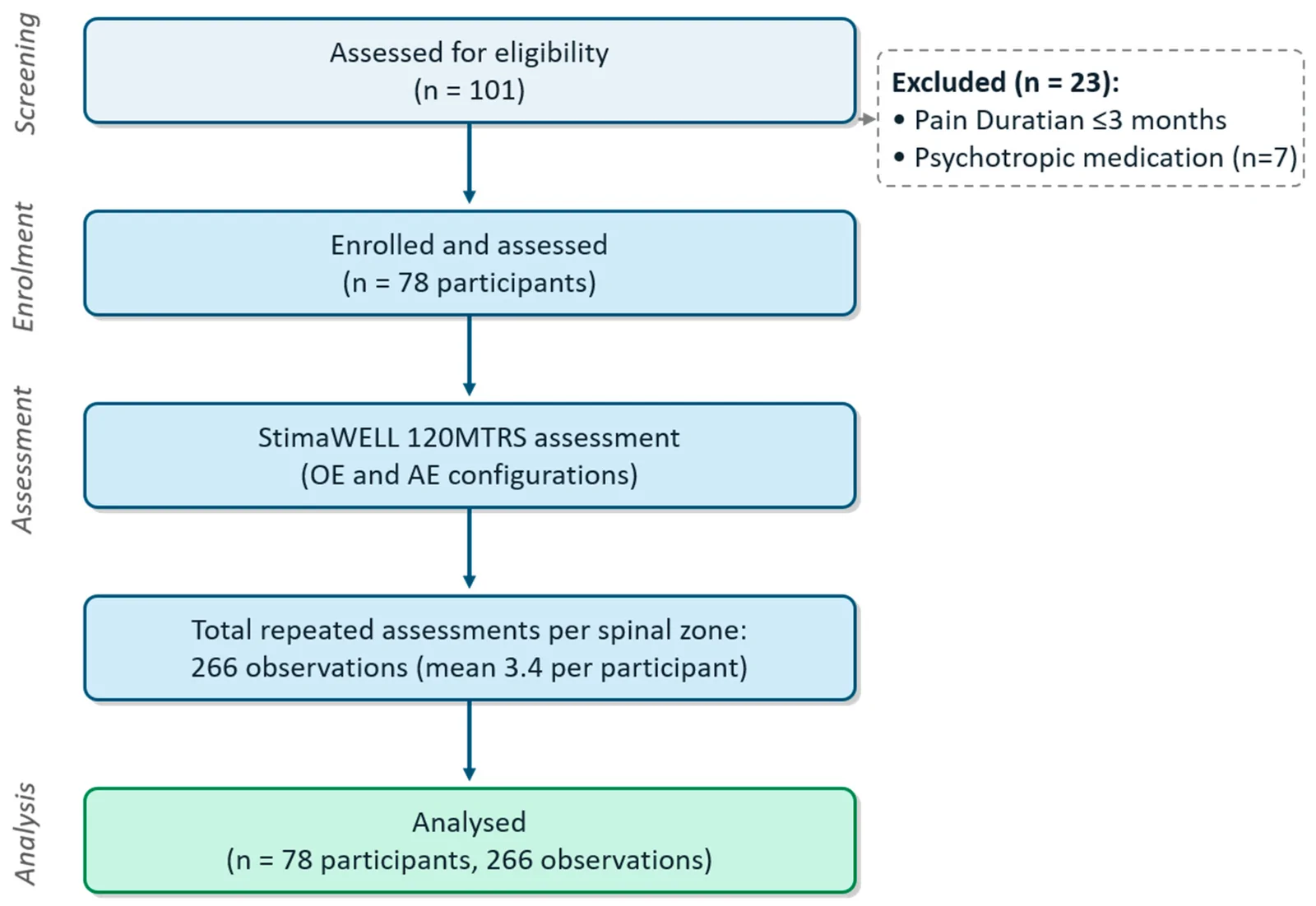
Participant flow diagram.

A convenience sample of 78 adults having spinal pain was recruited from a private physiotherapy clinic in Amasya, Türkiye. The volunteered participants were included in this study if they met the following criteria: (a) aged 18 years or older; (b) having diagnosed back pain by a physician; (c) experiencing chronic pain (duration > 3 months) with a relapse within the past 7 days; (d) no recent use of an analgesic and myorelaxant; (e) being literate; and (f) able to cooperate. Participants had a history of neurological, inflammatory, or psychiatric disorders requiring medication; cognitive, vestibular, or auditory impairments; undiagnosed acute-onset pain, radiculopathy, severe spinal osteoporosis, or a history of vertebral fractures; presence of electronic implants, cardiac arrhythmias, or coronary/carotid stents; active skin lesions or dermatological conditions at the electrode application sites; and were pregnancy or suspicion of pregnancy were excluded (Figure 1).

### 2.3. Data Collection

After recording the ages, genders, weights and heights of the participants, the location and intensity of pain were investigated. The participants were also assessed via the StimaWELL 120 MTRS system.

#### 2.3.1. Pain Assessment

**Figure 2:**
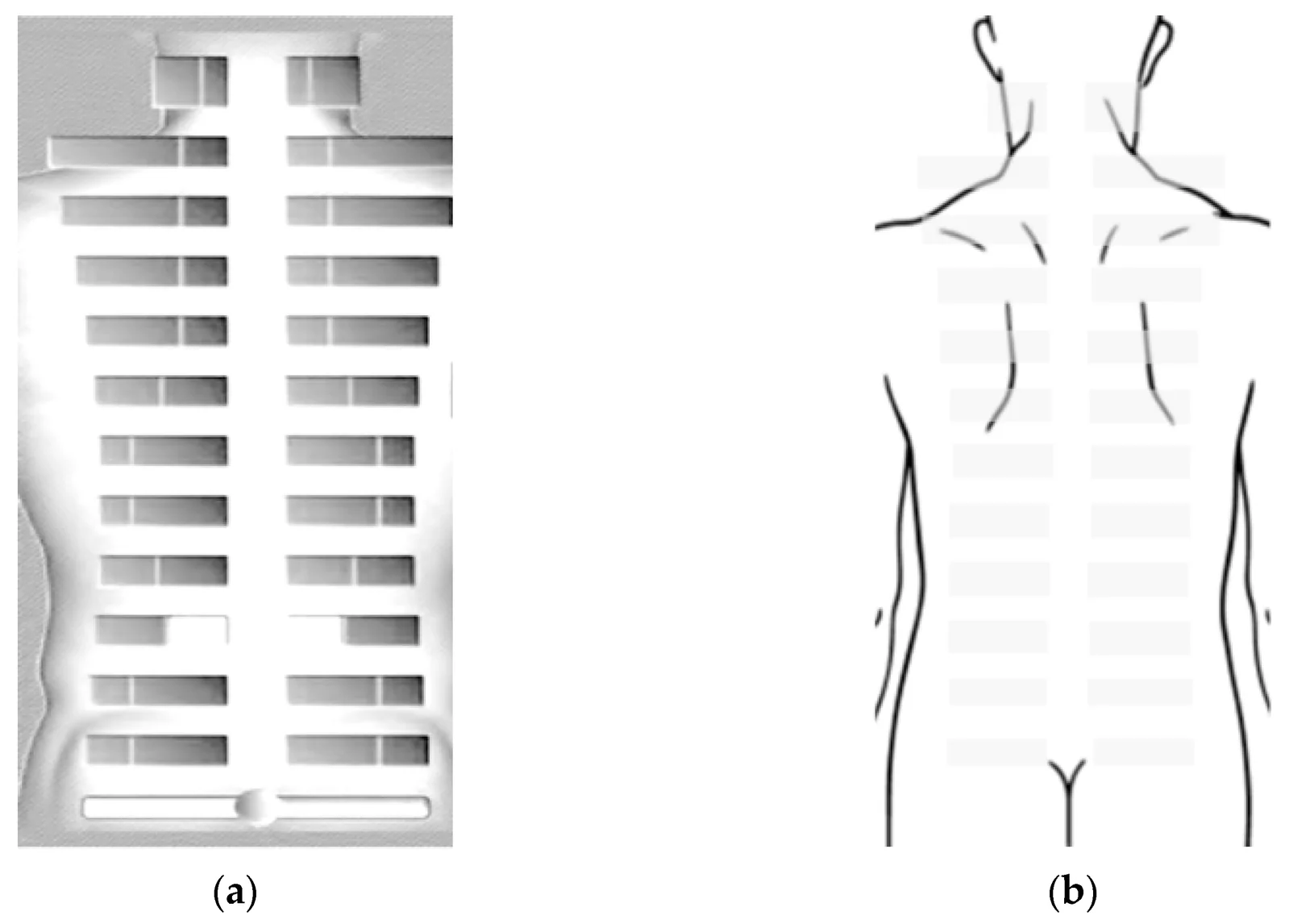
(**a**) The electrode mapping derived from the handbook of the device. (**b**) The body diagram integrated with electrode measurement zones (developed by the researchers).

The participants were asked to mark all the painful sites that they experienced within the last week on a body diagram (Figure 2). On this diagram, the areas corresponding to the 12 electrode channels of the device (detailed in Section 2.3.2) were faintly indicated and numbered 1 to 12 from top to bottom. For each painful site, patients were asked to report their pain intensity according to the Numerical Pain Rating Scale (NPRS).

The NPRS is an 11-point, simple, reliable, and rapidly administered scale frequently utilized in clinical settings to measure pain intensity [10]. The participants were instructed to rate their pain intensity for each location on a scale where “0” represents the absence of pain and “10” denotes the most severe pain imaginable on the body diagram.

#### 2.3.2. StimaWELL 120 MTRS

The StimaWELL 120MTRS (schwa-medico Medizinische Apparate Vertriebsgesellschaft mbH, Ehringshausen, Germany) is an AI-assisted spinal rehabilitation device using modulated medium-frequency stimulation, which is directed by a wave-mat with 12 channels [10,11,13,14]. These channels were numbered 1 to 12 from top to bottom, serving as the basis for the zones on the pain assessment body diagram. Participants lay on a paper towel that had been sprayed with warm water and spread over the wave mat. A drape was provided to ensure patient privacy. Participants were asked to remove their upper clothing (if appreciable) and position their lumbar vertebrae within the curved region of the device, with the spinous processes aligned between the electrodes. They were asked to lower the upper section of their undergarments so that the coccyx contacts the lowest channel. A triangular positioning pillow was placed under their knees to maximize surface contact with the channels in a relaxed position.

Before the rehabilitation protocol, the device performs a calibration protocol to standardize the stimulation intensity perceived by the patient through the wave-mat, as described in Balthazard et al. [10]. During this protocol, the stimulation intensity delivered by the device was adjusted until participants perceived a “strong but non-painful sensation,” which was recorded as the outcome measure. This calibration protocol consisted of three distinct phases. In the first phase, a general measurement was performed as the current moved repetitively along the full length of the wave-mat in a bottom–up direction. In the second phase, the current was adjusted sequentially from bottom to top for each pair of opposing channels to ensure that each pair reached the strong but non-painful threshold (yielding 12 outcomes). In the third and final phase, the current was delivered adjacent in two-channel groups (moving from bottom to top) to guarantee uniform intensity from one side to the other (yielding another 12 outcomes).

### 2.4. Data Preparation and Statistical Modeling

The clinical, anthropometric, and clinical data derived from the participants were structured for multivariate predictive analyses and managed as follows.

For the first stage of analysis, repeated measures correlation (rmcorr) was calculated to examine the linear association between each StimaWELL 120MTRS configuration and NPRS. Unlike conventional Pearson correlation, rmcorr accounts for the non-independence of repeated observations nested within participants, making it appropriate for datasets with multiple anatomical-site measurements per individual [20]. Intraclass correlation coefficients (ICC) were subsequently calculated to quantify absolute agreement between device-derived scores and NPRS. ICC values were interpreted according to established benchmarks: values below 0.50 indicate poor agreement, 0.50–0.75 moderate agreement, 0.75–0.90 good agreement, and above 0.90 excellent agreement [21].

In the second stage, linear mixed models (LMM) were executed to evaluate the associations between StimaWELL 120MTRS scores and NPRS while accounting for the repeated-measures structure of the data. Participant identity was included as a random effect to model within-person correlation across repeated anatomical-site measurements. Demographic and anthropometric variables (age, gender, height, weight) were entered as fixed-effect covariates alongside stimulation intensities from both electrode configurations.

For the last stage, the dataset was deployed into ML pipelines. All analyses were performed in Python (version 3.13.16) using scikit-learn. Missing data were handled via listwise deletion. To prevent data leakage in the context of repeated measures, dataset partitioning was performed at the person level (ID-based split): 80% of participants were allocated to the training set and 20% to the test set, ensuring that no participant’s observations appeared in both sets simultaneously. Given the relatively small sample size, extensive hyperparameter tuning and feature scaling were omitted; all algorithms were run with robust default settings (e.g., n_estimators = 100 for tree-based models) to reduce the risk of overfitting. Eight algorithms were evaluated: Extra Trees Regressor, Random Forest, Gradient Boosting, Hist Gradient Boosting, XGBoost, LightGBM, CatBoost, and Ridge Regression. Model performance was assessed using R^2^, Mean Absolute Error (MAE), Root Mean Squared Error (RMSE), and tolerance-based accuracy (TBA), defined as the proportion of predictions falling within a 10% error margin. Feature importance was calculated using the Mean Decrease in Impurity (MDI) metric for the three best-performing algorithms (Extra Trees, Gradient Boosting, CatBoost). The exact configuration of matched pain zones and electrodes is presented in Table 1.

## 3. Results

### 3.1. Participants

Seventy-eight volunteers (44 females and 34 males), aged 23–78 years, participated in the study. The participants had a mean height of 168.59 ± 9.10 cm and a mean body weight of 74.00 ± 16.54 kg (Table 2).

### 3.2. Correlation Analysis

**Figure 3:**
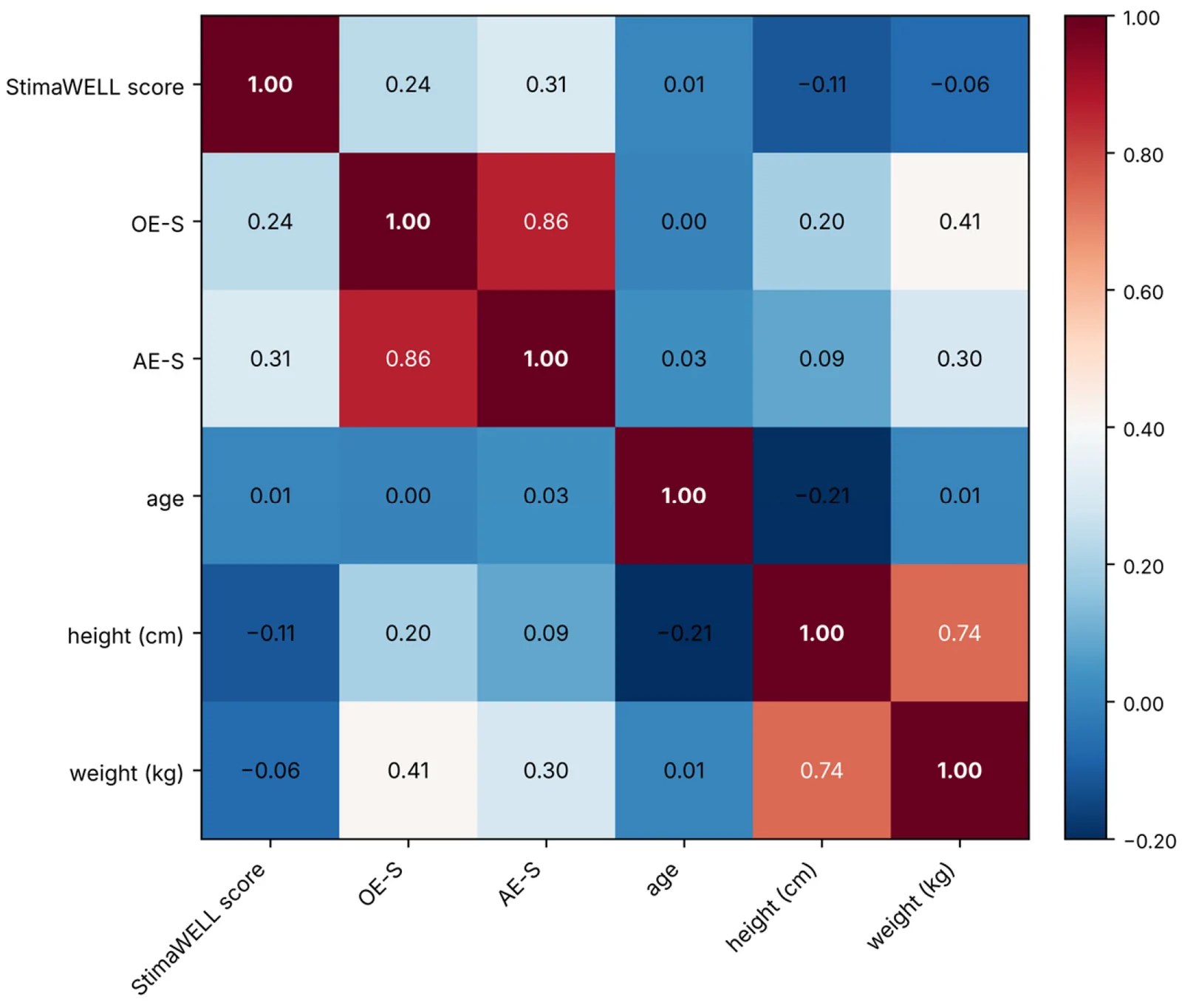
Correlation matrix of clinical and anthropometric variables (age, height, weight, OE-StimaWELL score, AE-StimaWELL score, and NPRS).

Repeated measures correlations (rmcorr) and intraclass correlation coefficients (ICC) between each StimaWELL 120MTRS configuration and NPRS are presented in Table 3. For the OE-StimaWELL configuration, the repeated measures correlation was negligible and non-significant (r = −0.003, *p* = 0.970). The ICC for OE-StimaWELL versus NPRS was 0.364 (95% CI: 0.19–0.50; F = 1.647; *p* < 0.001), indicating poor agreement. For the AE-StimaWELL configuration, the repeated measures correlation was similarly negligible (r = 0.015, *p* = 0.876). The ICC for AE-StimaWELL versus NPRS was 0.452 (95% CI: 0.31–0.57; F = 1.824; *p* < 0.001), indicating moderate absolute agreement. Figure 3 presents the correlation matrix of clinical and anthropometric variables, illustrating pairwise relationships among age, height, weight, OE-StimaWELL score, AE-StimaWELL score, and NPRS, and visually confirming the weak linear associations between device-derived scores and self-reported pain intensity.

### 3.3. Linear Mixed Model

Two separate linear mixed models were constructed to examine the extent to which StimaWELL 120MTRS scores predicted self-reported NPRS, after adjusting for the covariates age, gender, height, and weight. Model 1 incorporated the OE StimaWELL score, and Model 2 incorporated the AE StimaWELL score as the primary predictor of interest. In both models, gender was coded as 1 = female and 2 = male.

#### 3.3.1. Model 1: Opposing Electrode StimaWELL Score

The first linear mixed model, which included the OE-StimaWELL score alongside age, gender, height, and weight as predictors of NPRS, did not demonstrate a statistically significant overall association. The OE-StimaWELL score was not a significant predictor of NPRS (B = 0.068, SE = 0.087, *p* = 0.434). Among the demographic covariates, age (B = −0.008, SE = 0.027, *p* = 0.756), gender (B = −1.649, SE = 0.063, *p* = 0.879), height (B = 0.010, SE = 0.029, *p* = 0.386), and weight (B = 0.025, SE = 1.032, *p* = 0.115) were also non-significant.

#### 3.3.2. Model 2: Adjacent Electrode StimaWELL Score

The second linear mixed model, which included the AE-StimaWELL score alongside the same demographic covariates, also did not reveal statistically significant associations. The AE-StimaWELL score was not a significant predictor of NPRS (B = 0.098, SE = 0.087, *p* = 0.259). Demographic covariates including age (B = −0.009, SE = 0.027, *p* = 0.740), gender (B = −1.648, SE = 1.012, *p* = 0.108), height (B = 0.011, SE = 0.063, *p* = 0.852), and weight (B = 0.023, SE = 0.028, *p* = 0.403) were also non-significant.

Full model coefficients for both models are presented in Table 4.

### 3.4. Machine Learning

Eight ML algorithms (Extra Trees Regressor, Random Forest, Gradient Boosting, Hist Gradient Boosting, XGBoost, LightGBM, CatBoost and Ridge Regression) were used to predict self-reported pain intensity (NPRS) from StimaWELL 120MTRS scores and demographic/anthropometric variables. Separate models were developed for the OE and AE configurations. Model performance was assessed using the coefficient of determination (R^2^), mean absolute error (MAE), root mean square error (RMSE), and tolerance-based accuracy (TBA). Feature importance was also calculated to identify the relative contribution of each predictor.

#### 3.4.1. Model Performance

Across both electrode configurations, the ML models demonstrated modest and variable predictive performance for NPRS, with overall R^2^ values suggesting limited explanatory power in many algorithms (Table 5a,b). For the OE-StimaWELL models, Extra Trees achieved the highest R^2^ (19.53 ± 11.65%), with an MAE of 1.21 ± 0.25 and RMSE of 1.65 ± 0.36, and a tolerance-based accuracy (TBA) of 66.51 ± 4.83%. Ridge Regression and CatBoost also produced positive R^2^ values (14.87 ± 21.19% and 11.24 ± 33.55%, respectively), while the remaining tree-based ensemble algorithms yielded negative R^2^ values relative to the mean baseline, indicating that the mean predictor outperformed these models on the test set for this configuration. For the AE-StimaWELL models, Extra Trees again performed competitively (R^2^ = 6.90 ± 33.06%; MAE = 1.06 ± 0.22; RMSE = 1.51 ± 0.35; TBA = 73.64 ± 9.00%), alongside CatBoost (TBA = 73.67 ± 9.66%) and Gradient Boosting (TBA = 73.27 ± 10.89%). Notably, AE-based models showed higher TBA values overall compared to OE-based models, suggesting that the AE configuration may carry marginally more predictive information for self-reported pain intensity. The mean baseline model performed consistently worse across all configurations, confirming the relative utility of the ML approaches despite modest absolute performance.

**Figure 4:**
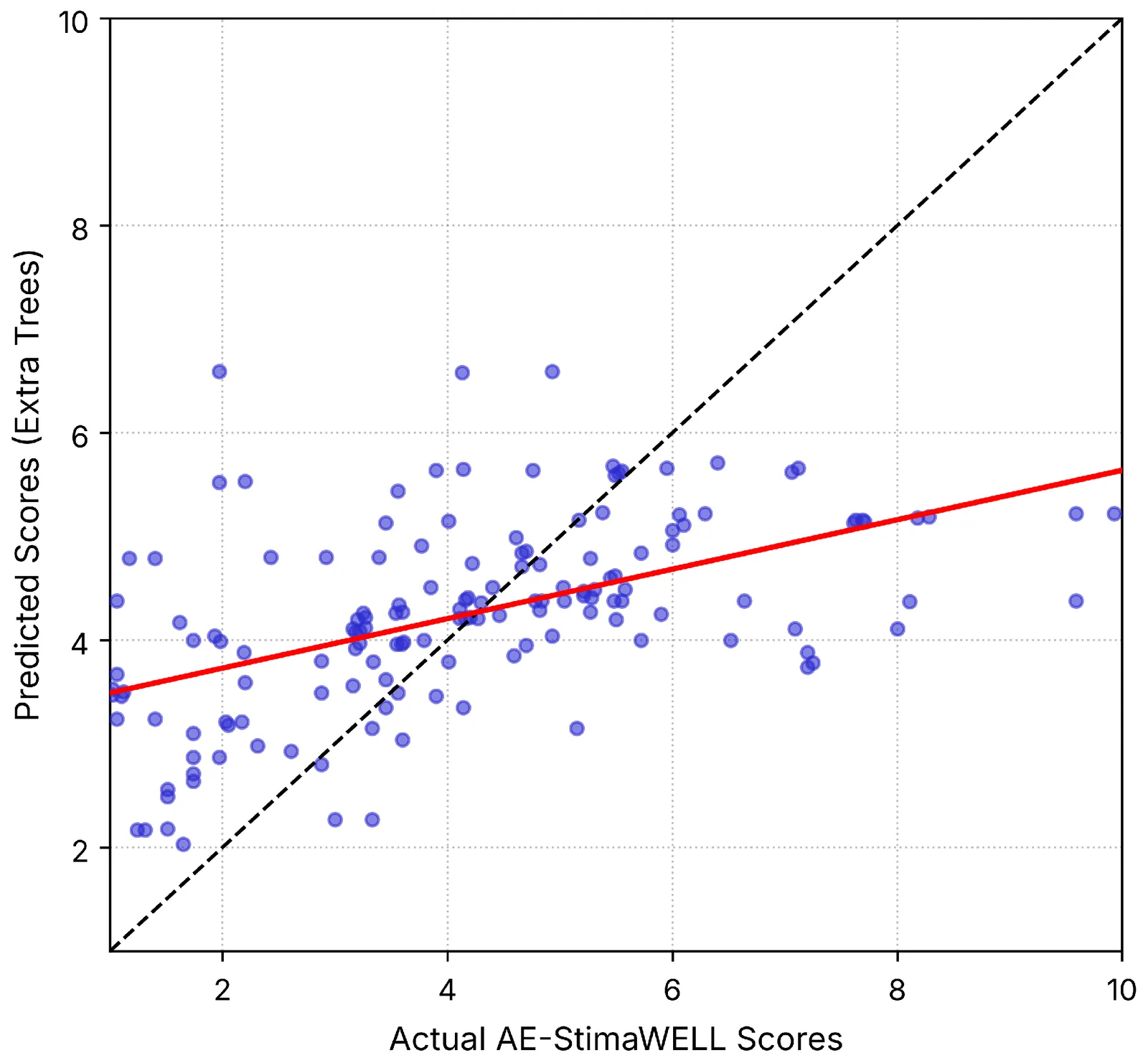
Scatter plot of actual versus predicted NPRS values for the best-performing machine learning model (Extra Trees, AE-StimaWELL configuration). Each point represents one observation. The dashed line indicates perfect agreement between actual and predicted values (identity line, slope = 1). The red line represents the linear regression fit between actual and predicted scores, with the shaded area indicating the 95% confidence interval.

Figure 4 presents a scatter plot of actual versus predicted NPRS values for the selected ML model, providing a visual assessment of model fit and prediction error distribution across the observed pain score range.

#### 3.4.2. Feature Importance

Feature importance scores for the three best-performing algorithms, Extra Trees (ET), Gradient Boosting (GB), and CatBoost, are presented in Table 6 for both electrode configurations. Across all models, the StimaWELL device score was the single most important predictor, contributing 22–28% of the predictive weight in OE-based models and 32–37% in AE-based models. Among the remaining variables, weight and height were the next most influential predictors across both configurations, contributing approximately 17–36% and 17–21%, respectively. Age contributed moderately across all models (16–22%), while gender showed the most variable importance, ranging from 6% (GB, OE) to 17% (CatBoost, OE), and contributing less in AE-based models overall. The AE-StimaWELL score consistently showed higher feature importance than the OE score across all three algorithms.

## 4. Discussion

This study examined the relationship between StimaWELL 120MTRS-derived stimulation-threshold measurements and self-reported pain intensity (NPRS) in individuals with spinal pain in a clinical sample of 78 adults. The findings from repeated measures correlation, intraclass correlation, linear mixed modeling, and machine learning analyses collectively indicate that the StimaWELL 120MTRS stimulation thresholds, measured in both OE and AE configurations, do not demonstrate strong linear associations with patient-reported pain intensity, and their predictive value in isolation and in combination with demographic variables remains limited.

The repeated measures correlations between both StimaWELL configurations and NPRS were negligible and statistically non-significant (r = −0.003, *p* = 0.970 for OE; r = 0.015, *p* = 0.876 for AE), indicating that variation in stimulation thresholds across repeated assessments does not track with variation in self-reported pain scores at the within-person level. The ICC values, while statistically significant (OE: ICC = 0.364, *p* < 0.001; AE: ICC = 0.452, *p* < 0.001), fell in the poor range, suggesting only limited absolute agreement between device-derived and patient-reported measures. This pattern is consistent with the conceptual distinction between nociceptive threshold, as captured by the device’s calibration protocol, and the subjective, multidimensional experience of pain intensity [5,7]. Stimulation tolerance may reflect local muscle recruitment capacity, tissue impedance, and cortical habituation, each of which can vary independently of perceived pain [4,12].

The linear mixed models confirmed the absence of statistically significant associations between either the StimaWELL configuration and the NPRS after adjusting for age, gender, height, and weight. None of the demographic covariates reached significance in either model, which may partly reflect the relatively homogeneous clinical profile of the sample and the cross-sectional, single-session assessment design. The absence of a weight effect is noteworthy given published evidence linking adiposity to spinal pain burden [22,23,24]; however, the present sample’s range of body weight (44–115 kg) and the confounding influence of pain location heterogeneity across the cervical, thoracic, lumbar, and sacral zones may have attenuated this relationship.

The machine learning analyses extended the regression findings by exploring non-linear interactions among predictors. Despite employing eight diverse algorithms, spanning regularized linear methods (Ridge Regression) to advanced gradient-boosting frameworks (XGBoost, LightGBM, CatBoost) and tree-based ensembles (Extra Trees, Random Forest, Gradient Boosting, Hist Gradient Boosting), the predictive performance was modest. The best-performing algorithms, Extra Trees and CatBoost, achieved tolerance-based accuracies of approximately 66–74%, with R^2^ values rarely exceeding 20%. Notably, AE-based models consistently outperformed OE-based models across most algorithms, suggesting that the AE configuration may carry marginally more biologically relevant signal for pain prediction.

Despite the absence of statistically significant associations in the linear mixed models, the ML algorithms achieved meaningful predictive performance as assessed by TBA, with the best-performing models correctly predicting NPRS within a 10% error margin in up to 73–74% of cases. The 10% tolerance threshold adopted in this study was defined a priori based on the 0–10 NPRS scale, corresponding to a maximum acceptable prediction error of 1 point, a margin broadly consistent with the minimal clinically important difference (MCID) estimates reported for spinal pain populations. It should be noted, however, that MCID estimates vary substantially across studies and populations; a review of relative MCID values found median estimates around 32–34% with considerable heterogeneity [25], and a frequently cited benchmark of approximately 30% reduction in pain intensity reflects a threshold for clinically meaningful patient improvement rather than an acceptable model prediction error [26,27]. These distinctions are important because TBA, as reported here, reflects the proportion of predictions falling within a prespecified absolute error band, not a claim about clinical response thresholds. Comparison with published pain-prediction studies further illustrates this challenge. An EEG-based model reported mean absolute errors (MAEs) of 19–21 points on a 0–100 scale yet still failed to outperform a simple mean baseline [28], while a spinal pain study reported estimation errors of 16.5–32.1% [29]. These examples highlight that no agreed standard for acceptable prediction error exists in this field, reinforcing the importance of reporting MAE and RMSE alongside a naive baseline for meaningful interpretation.

An unexpected finding was that the newly added advanced gradient-boosting algorithms (XGBoost, LightGBM, CatBoost) did not outperform the classical ensemble methods (Extra Trees, Random Forest), where modern boosting frameworks generally demonstrate superior performance [30]. This may be explained by the relatively small sample size of the present study: advanced gradient-boosting algorithms are known to be more sensitive to overfitting when training data are limited, and their performance advantage over simpler ensembles tends to emerge only with larger datasets [31,32]. Importantly, to mitigate overfitting and prevent data leakage, the dataset in this study was partitioned at the participant level rather than at the observation level, ensuring that all observations from a given participant appeared exclusively in either the training or the test set, never both. This person-level split is methodologically more rigorous than a random observation-level split in repeated-measures designs, as the latter would allow the model to learn participant-specific patterns during training and artificially inflate test performance [33].

The StimaWELL device score was the most important predictor across all three algorithms (ET, GB, CatBoost) and both electrode configurations (OE: 22–28%; AE: 32–37%). This finding supports the device’s potential as a quantitative input within a multivariate pain prediction framework, even in the absence of strong linear associations with NPRS. The consistently higher feature importance of the AE score compared to the OE score across all algorithms aligns with the marginally superior predictive accuracy of AE-based models, suggesting that the AE configuration may capture a greater proportion of the variance in self-reported pain intensity.

Weight and height were the next most influential predictors, each contributing approximately 17–36% and 17–21%, respectively. The prominence of weight is consistent with evidence linking higher BMI to greater spinal pain burden [34,35] through mechanical loading and low-grade inflammation [36,37]. A similar pattern of weight prominence was reported by Zhang et al. [22], who identified BMI and weight-adjusted waist index among the most important features in a Random Forest model predicting chronic low back pain, and by Wu et al. [23], who demonstrated that BMI was among the most influential predictors across multiple ML models in a large retrospective cohort of 67,028 outpatient cases. The notably high weight importance in the Gradient Boosting OE model (36.21%) should be interpreted with caution, as it was markedly higher than in the ET and CatBoost models, potentially reflecting algorithm-specific sensitivity. Although weight is widely used in clinical practice, emerging evidence suggests that central adiposity may be a stronger predictor of pain than BMI alone [24]. Future studies should therefore incorporate measures such as waist circumference or A Body Shape Index (ABSI) alongside conventional anthropometric variables to better characterize the anthropometric predictors of spinal pain intensity. Age contributed moderately across models (16–22%), while gender showed the most variable importance (6–17%), possibly reflecting differences in how algorithms handle categorical variables rather than a true biological effect.

Finally, the challenge of data collection in clinical populations with spinal pain, which requires repeated device assessments across multiple sessions, underscores the need for ML systems capable of delivering reliable predictions from small samples. Future methodological work should prioritize the development of sample-efficient learning frameworks, such as transfer learning or few-shot approaches, that can be validly applied in resource-constrained clinical research settings.

### 4.1. Clinical Implications

The findings suggest that StimaWELL 120MTRS-derived stimulation thresholds carry predictive information within a multivariate framework, consistently emerging as the most important predictor across all ML algorithms. However, the absence of significant associations in correlation and linear mixed model analyses indicates that device scores and self-reported pain intensity are not interchangeable. Clinicians should therefore interpret StimaWELL output as complementary to patient-reported measures such as the NPRS, not as a substitute.

The modest overall ML performance reflects the multidimensional nature of spinal pain, which extends beyond the physiological and anthropometric variables captured here. The AE configuration consistently outperformed the OE configuration, suggesting that it may yield marginally more informative measurements in clinical practice.

Body weight was a consistently influential predictor across models. Clinicians should account for body weight when interpreting device output, as higher stimulation thresholds may partly reflect mechanical loading rather than pain-specific dysfunction, and weight management may enhance StimaWELL-guided rehabilitation outcomes.

### 4.2. Strengths, Limitations and Future Directions

This study employed both linear mixed modeling and machine learning, combining the inferential strengths of statistical approaches with the flexibility of ML to capture non-linear relationships among predictors [30]. The use of repeated-measures correlation and person-level train/test splitting are methodological strengths appropriate for the repeated-measures design, reducing the risk of data leakage and overfitting. The inclusion of eight diverse algorithms across two electrode configurations provided a comprehensive evaluation of predictive utility.

Several limitations should be acknowledged. The cross-sectional design precludes causal inference, and future longitudinal studies are needed to examine whether StimaWELL measurements track clinically meaningful changes in pain over time. The relatively small sample size may have limited model stability, and the absence of significant associations in both correlation and regression analyses may partly reflect insufficient statistical power. Only demographic and anthropometric variables were included; psychological factors, pain duration, physical function, and imaging findings, all established pain determinants, were not assessed. Body weight was measured without indices of central adiposity, which may better characterize obesity-related pain risk [24]. Finally, while the ML models demonstrated acceptable tolerance-based accuracy, clinical trustworthiness and generalizability of ML algorithms in healthcare remain a recognized concern [31,32,38]. Therefore, external validation in larger, more diverse populations is required before these models can be considered for routine clinical use. Future studies should incorporate broader biopsychosocial variables, longitudinal designs, and larger samples to improve both predictive performance and clinical applicability. Furthermore, body awareness and pain localization may differ across spinal regions, and evidence suggests that tactile acuity and body schema are altered in individuals with chronic spinal pain [39,40,41]. Although measurements were matched to their corresponding anatomical zones, as detailed in Table 1, pooling observations across cervical, thoracic, lumbar, and sacral regions may have introduced heterogeneity in the outcome measures that could not be fully controlled in the current analyses.

## 5. Conclusions

This study investigated whether StimaWELL 120MTRS-derived stimulation thresholds could predict self-reported spinal pain intensity using repeated measures correlation, linear mixed modeling, and ML. Neither electrode configuration demonstrated significant linear associations with NPRS, and the linear mixed models did not identify device scores or demographic variables as significant independent predictors. Despite this, ML algorithms achieved moderate tolerance-based accuracy, with the AE configuration consistently outperforming the OE configuration across both statistical and ML analyses. Across all models, the StimaWELL device score was the single most important predictor, followed by body weight and height, underscoring the relevance of anthropometric context when interpreting device output.

These findings suggest that StimaWELL 120MTRS stimulation thresholds may contribute quantitative information within a multivariate pain assessment framework but should not be used as a standalone substitute for patient-reported outcomes. The small sample size, single-center design, and absence of psychosocial variables limit the generalizability of these findings. Future studies should validate these models in larger and more diverse populations, incorporate broader clinical and biopsychosocial variables, and employ longitudinal designs to assess whether device-derived measurements track clinically meaningful changes in pain over time.

## Figures and Tables

**Table 1:** Anatomical mapping of electrode pairs and corresponding stimulation intensities: opposing vs. adjacent configurations.

Spinal Region	Zones of Electrode Pairs	Opposing Measurement	Adjacent Measurement
Cervical	1st and 2nd pairs (R)	1st and 2nd pairs	1st and 2nd pairs (R)
Cervical	1st and 2nd pairs (L)	1st and 2nd pairs	1st and 2nd pairs (L)
Thoracic	3rd through 8th pairs (R)	3rd through 8th pairs	3rd through 8th pairs (R)
Thoracic	3rd through 8th pairs (L)	3rd through 8th pairs	3rd through 8th pairs (L)
Lumbar	9th and 10th pairs (R)	9th and 10th pairs	9th and 10th pairs (R)
Lumbar	9th and 10th pairs (L)	9th and 10th pairs	9th and 10th pairs (L)
Sacral	11th and 12th pairs (R)	11th and 12th pairs	11th and 12th pairs (R)
Sacral	11th and 12th pairs (L)	11th and 12th pairs	11th and 12th pairs (L)

R = right; L = left.

**Table 2:** Characteristics of participants (*n* = 78).

Variables	Mean ± s.d.	Min–Max
Age	43.08 ± 11.50	23–78
Height (cm)	168.59 ± 9.10	147–184
Weight (kg)	74.0 ± 16.54	44–115
Gender	n	%
Female	44	56.4
Male	34	43.6

s.d. = standard deviation; n = number; % = percentage.

**Table 3:** Repeated Measures Correlations and Intraclass Correlation Coefficients Between StimaWELL 120MTRS Scores and Self-Reported Pain Intensity (NPRS).

Variable Pair	r	*p* (rmcorr)	ICC	*p* (ICC)	95% CI	F
NPRS vs. OE-StimaWELL	−0.003	0.970	0.364	<0.001	0.19–0.50	1.647
NPRS vs. AE-StimaWELL	0.015	0.876	0.452	<0.001	0.31–0.57	1.824

NPRS = Numeric Pain Rating Scale; OE = opposing-electrode StimaWELL score; AE = adjacent-electrode StimaWELL score; r = repeated measures correlation coefficient; *p* (rmcorr) = significance value for repeated measures correlation; ICC = intraclass correlation coefficient; *p* (ICC) = significance value for ICC; CI = confidence interval; F = F-statistic. ICC benchmarks: <0.50 poor, 0.50–0.75 moderate, 0.75–0.90 good, >0.90 excellent agreement.

**Table 4:** Linear Mixed Model Coefficients for Models 1 (OE) and 2 (AE) Predicting NPRS.

Predictor	Model 1: OE-StimaWELL Score			Model 2: AE-StimaWELL Score		
	**B**	**SE**	***p***	**B**	**SE**	***p***
Constant	4.420	9.151	0.631	4.114	9.075	0.652
StimaWELL score	0.068	0.087	0.434	0.098	0.087	0.259
Age (years)	−0.008	0.027	0.756	−0.009	0.027	0.740
Gender	−1.649	0.063	0.879	−1.648	1.012	0.108
Height (cm)	0.010	0.029	0.386	0.011	0.063	0.852
Weight (kg)	0.025	1.032	0.115	0.023	0.028	0.403

OE = opposing-electrode StimaWELL score; AE = adjacent-electrode StimaWELL score; B = unstandardized coefficient; SE = standard error; *p* = significance value. Gender coded 1 = female, 2 = male.

**Table 5:** (**a**). Machine Learning Algorithm Performance for Predicting NPRS—OE-StimaWELL Models. (**b**). Machine Learning Algorithm Performance for Predicting NPRS—AE-StimaWELL Models.

(**a**)				
**Algorithm**	**OE-StimaWELL Models**			
	**R ^2^ (%)**	**MAE**	**RMSE**	**TBA (%)**
Mean (Reference)	−2.78 ± 2.78	1.50 ± 0.42	1.88 ± 0.43	53.69 ± 13.98
Ridge Regression	14.87 ± 21.19	1.24 ± 0.13	1.65 ± 0.20	64.70 ± 5.87
Random Forest	2.66 ± 26.18	1.32 ± 0.24	1.78 ± 0.34	63.54 ± 5.39
Extra Trees	19.53 ± 11.65	1.21 ± 0.25	1.65 ± 0.36	66.51 ± 4.83
Gradient Boosting	−4.58 ± 46.69	1.30 ± 0.18	1.79 ± 0.30	63.54 ± 6.96
Hist Gradient Boosting	−10.34 ± 41.28	1.41 ± 0.35	1.87 ± 0.45	62.37 ± 10.36
XGBoost	−26.89 ± 57.83	1.56 ± 0.46	2.02 ± 0.60	55.58 ± 9.54
LightGBM	−11.61 ± 43.10	1.40 ± 0.27	1.87 ± 0.35	61.25 ± 8.16
CatBoost	11.24 ± 33.55	1.25 ± 0.18	1.68 ± 0.29	65.76 ± 6.37
(**b**)				
**Algorithm**	**AE-StimaWELL Models**			
	**R ^2^ (%)**	**MAE**	**RMSE**	**TBA (%)**
Mean (Reference)	−7.86 ± 11.70	1.31 ± 0.40	1.69 ± 0.50	64.25 ± 12.01
Ridge Regression	−2.58 ± 30.42	1.21 ± 0.21	1.58 ± 0.31	65.00 ± 8.09
Random Forest	−2.05 ± 59.79	1.05 ± 0.14	1.49 ± 0.22	71.01 ± 9.44
Extra Trees	6.90 ± 33.06	1.06 ± 0.22	1.51 ± 0.35	73.64 ± 9.00
Gradient Boosting	−18.27 ± 81.20	1.09 ± 0.17	1.57 ± 0.25	73.27 ± 10.89
Hist Gradient Boosting	−13.33 ± 37.17	1.20 ± 0.34	1.67 ± 0.40	70.24 ± 13.53
XGBoost	−23.47 ± 72.03	1.20 ± 0.32	1.65 ± 0.42	66.89 ± 12.32
LightGBM	−12.86 ± 41.34	1.17 ± 0.28	1.64 ± 0.34	68.76 ± 9.22
CatBoost	−6.98 ± 49.07	1.10 ± 0.20	1.59 ± 0.34	73.67 ± 9.66

R^2^ = coefficient of determination; MAE = mean absolute error; RMSE = root mean squared error; TBA = tolerance-based accuracy (10% error margin); OE = opposing-electrode StimaWELL score; AE = adjacent-electrode StimaWELL score. Dependent variable: NPRS. Predictors in all models: OE / AE StimaWELL score, age, gender, height, weight.

**Table 6:** Feature Importance Scores (%) for Ensemble Models—OE- and AE-StimaWELL Score.

Predictor	OE-StimaWELL Models			AE-StimaWELL Models		
	ET	GB	CatBoost	ET	GB	CatBoost
StimaWELL score	27.80	22.27	25.87	37.20	36.24	31.97
Weight (kg)	19.40	36.21	19.21	19.16	15.32	16.67
Height (cm)	20.62	17.41	20.29	18.49	19.54	19.72
Age (years)	16.40	18.41	17.18	17.07	21.99	19.53
Gender	15.78	5.60	17.45	8.08	6.71	12.11

Values represent each predictor’s percentage contribution to the model’s predictive power. OE = opposing-electrode StimaWELL score; AE = adjacent-electrode StimaWELL score; ET = Extra Trees; GB = Gradient Boosting. Feature importance was calculated using the Mean Decrease in Impurity (MDI) metric.

## Data Availability

The data presented in this study are available from the corresponding author upon reasonable request. The data are not publicly available due to ethical and privacy considerations related to human participant data.

## Supplementary Materials

The following supporting information can be downloaded at https://www.mdpi.com/article/10.3390/diagnostics16203403/s1, Checklist S1: STROBE Checklist for Cross-Sectional Studies.

## Abbreviations

The following abbreviations are used in this manuscript:

AI	Artificial Intelligence
AE	Adjacent Electrode
ABSI	A Body Shape Index
BMI	Body Mass Index
ICC	Intraclass Correlation Coefficients
LMM	Linear Mixed Model
MAE	Mean Absolute Error
MCID	Minimal Clinically Important Difference
MDI	Mean Decrease in Impurity
ML	Machine Learning
MTRS	Multimodal Therapy and Relief System
NPRS	Numeric Pain Rating Scale
OE	Opposing Electrode
QST	Quantitative Sensory Testing
rmcorr	Repeated Measures Correlation
RMSE	Root Mean Squared Error
sEMG	Surface Electromyography
STROBE	Strengthening the Reporting of Observational Studies in Epidemiology
TBA	Tolerance-Based Accuracy
